# Psychometric Properties of the Adjustment to Aging Scale (Atas) in Iranian Older Adults

**DOI:** 10.3390/geriatrics10060149

**Published:** 2025-11-10

**Authors:** Parisa Mollaei, Yadollah-Abolfathi Momtaz, Malihe Saboor, Nasibeh Zanjari

**Affiliations:** Iranian Research Center on Aging, University of Social Welfare and Rehabilitation Sciences, Tehran 1985713834, Iran; par.mollaei@uswr.ac.ir (P.M.); ma.saboor@uswr.ac.ir (M.S.); na.zanjari@uswr.ac.ir (N.Z.)

**Keywords:** adjustment to aging, psychometrics properties, older adults, scale validation, Iranian population

## Abstract

**Background/Objectives:** Adjustment to aging is a key indicator of positive aging and psychological maturity, influenced by cultural and social contexts. This study aimed to translate and evaluate the psychometric properties of the Adjustment to Aging Scale (AtAS) among Iranian older adults. **Material and methods:** This cross-sectional study was conducted in Tehran, 2024. Following translation and cross-cultural adaptation, face validity, content validity, and reliability of the questionnaire were assessed. The WHO-5 well-being index was used to assess concurrent validity. A total of 328 older adults aged 60 years and above completed the study instruments. Data were analyzed using Confirmatory Factor Analysis (CFA), Cronbach’s alpha, Pearson correlation, independent t-tests, and ANOVA via SPSS version 22 and AMOS 24. The significance level was set at *p* ≤ 0.05. **Results:** The mean (SD) age of the participants was 69.42 (6.8) years. Face and content validity were confirmed by fourteen experts (CVI = 0.94). CFA supported the five-factor structure of the questionnaire (χ^2^/df = 2.06, GFI = 0.90, PCLOSE = 0.07, RMSEA = 0.05), indicating a good model fit. The total questionnaire showed acceptable internal consistency (Cronbach’s alpha = 0.80) and excellent test–retest reliability (ICC = 0.98). Pearson’s correlation revealed a significant positive relationship between the WHO-5 Well-Being Index and AtAS scores (r = 0.56, *p* < 0.05), supporting criterion validity. **Conclusions:** The Persian AtAS showed strong psychometric properties, supporting its use in both research and clinical settings, although further studies are recommended to strengthen evidence for its clinical application.

## 1. Introduction

The global population of older adults is increasing rapidly. According to the World Health Organization (WHO), in 2019, the number of individuals aged 60 years and above was one billion. This figure is projected to rise to 1.4 billion by 2030 and 2.1 billion by 2050 [1].

Aging brings about distinct changes, challenges, needs, and opportunities [2]. As people age, multiple dimensions of life—physical, psychological, social, financial, and occupational—undergo significant changes [3,4] and older adults face diminished initial control capacity, and personal and social resources [5].

Retirement as a significant milestone in the aging process, affecting daily routines, social connections, mental and physical health, and financial security [6]. Additionally, bereavement—particularly the loss of a spouse—can disrupt life stability and provoke prolonged periods of grief, stress, and fear [7,8]. Chronic diseases also become more prevalent with age, making older adults more vulnerable. Many experience depression, low self-esteem, reduced control over life aspects [9] and anxiety, particularly death anxiety [10]. Such challenges are exacerbated by changing family dynamics, such as the empty nest phenomenon [11] and an overall decline in functional capacities and independence [12]. Consequently, the ability to adjust to these evolving circumstances becomes a defining feature of aging [13] and serves as a foundational element of successful aging [14].

Adjustment to aging is a multidimensional concept that reflects how individuals respond to age-related changes. It entails the dynamic interaction of psychological resilience, social support, and adaptive behaviors. Beyond addressing physical limitations, this process encompasses the pursuit of meaning, purpose, and satisfaction in later life [15]. Adjustment to aging is largely achieved by maintaining equilibrium between personal experiences, standards, goals, motivations, and values, and the external circumstances encountered during later life [16,17].

Despite the availability of several instruments to assess adjustment to aging, including the Philadelphia Geriatric Center (PGC) Morale Scale [18], the Life Satisfaction Index [19], the General Adjustment to Aging Scale (GAAS) and the Perceived Social Support Scale [20], Bell’s Social Adjustment Scale [21,22], the Adjustment to Aging Scale (AtAS), and measures of subjective well-being [23], recent reviews indicate a lack of validated tools to assess adjustment to aging among Iranian older adults. Furthermore, existing tools often fail to fully capture the multidimensionality of adjustment in diverse socio-cultural contexts, highlighting a psychometric gap that necessitates rigorous translation and validation studies in local settings.

In previous studies, as physical, cognitive, and social roles evolve with age, so do the strategies for preserving well-being and adjusting to new realities. This necessitates a measurement approach that reflects the multidimensionality of adjustment [24]. Considering that prior research has highlighted the impact of cultural, national, and ethnic contexts on the aging process [25,26], the AtAS has gained recognition as a valuable cross-cultural tool for research, clinical practice, and program development in the field of healthcare [23].

Given that the applicability of measurement instruments is strongly influenced by socio-cultural context, it is necessary to evaluate the psychometric properties of tools developed elsewhere before their use in different populations. The AtAS was chosen for localization in Iran due to its comprehensiveness, assessing not only daily functioning and health but also broader aspects relevant to older adults’ well-being. Therefore, this study aimed to translate and validate the AtAS for use among Iranian older adults, providing an appropriate instrument for both research and clinical applications.

## 2. Materials and Methods

### 2.1. Study Design

This cross-sectional study was conducted in 2025 to translate and psychometrically validate the Adjustment to Aging Scale (AtAS) for use among Iranian older adults.

### 2.2. Participants

Since it is recommended to have 10–20 participants per item for factor analysis [27], a total of 330 older adults aged 60 years and above, residing in Tehran, Iran, were recruited, corresponding to 15 participants per each of the 22 questionnaire items. To ensure broad representation of the target population, the city of Tehran was divided into five areas based on socio-economic development levels [28]: developed, relatively developed, moderate development level, less developed, underdeveloped. one health center was randomly selected in each area. Older adults who attended these centers were invited to participate in the study. The number of participants from each area was calculated proportionally to the total number of older adults residing in that area to ensure that the study sample was representative of the population. The total number of older adults across the 22 areas of Tehran was 1104,834, with the following distribution in the selected areas: Area 2 (125,843), Area 4 (106,090), Area 5 (107,416), Area 14 (55,065), and Area 15 (58,398). After obtaining informed consent and confirming their willingness to participate, eligible individuals completed the questionnaire. Although maximum effort was made to ensure that the study sample was representative of the older adult population in Tehran, as a methodological limitation, it should be noted that the results primarily reflect the older adult population in Tehran, and generalization to the other older population should be made with caution.

### 2.3. Inclusion Criteria

Participants were eligible for the study if they met the following criteria: (1) aged 60 years or older; (2) willing to participate in the research; (3) Iranian nationality with the ability to understand and speak Persian; (4) capable of verbal communication and able to accurately respond to the questionnaire items; and (5) free from cognitive impairments. Exclusion Criteria: unwillingness to continue participation in the study and incomplete questionnaires.

### 2.4. Research Instrument

In this study, data were collected using a demographic information form, the Adjustment to Aging Scale (AtAS) and WHO 5 well-being index (for criterion validity).

a. Adjustment to Aging Scale (AtAS): This Scale (AtAS) was originally developed and psychometrically validated by Sofia von Humboldt et al. in 2014 [23]. AtAS was administered to 1291 community-dwelling older adults aged 75 to 102 years from both urban and rural areas across four nationalities (Angolan, Brazilian, English, and Portuguese). The AtAS is designed to measure the degree of adjustment to aging and consists of 22 items across five dimensions: Sense of Purpose and Ambition (SPA, 4 items), Zest and Spirituality (ZS, 5 items), Body and Health (BH, 5 items), Aging in Place and Stability (APS, 5 items), and Social Support (SS, 3 items). Items are rated on a 7-point Likert scale ranging from 1 (not important at all) to 7 (very important), with higher scores indicating greater adjustment to aging. The total score ranges from 22 to 154, and according to the original instrument and its author, the AtAS does not have a cut-off point. The internal consistency of the original scale was reported to be 0.89.
b. WHO 5 well-being index: This Index is a short, general measure developed by the World Health Organization to assess subjective well-being, focusing exclusively on positive statements [29]. The scale was first validated in Iran by Mortezavi et al. (2013), reporting a Cronbach’s alpha of 0.85 [30]. The scale consists of five items, each rated on a 6-point Likert scale based on how the respondent felt over the past two weeks. Response options range from “All of the time” (5) to “At no time” (0), with higher scores indicating greater well-being. The total raw score (ranging from 0 to 25) is multiplied by 4 to produce a final score between 0 and 100. A score above 52 is considered to indicate good well-being, whereas a score below 52 may reflect reduced well-being. Additionally, a raw score below 13 (before multiplication) may suggest poor emotional well-being and may warrant further assessment [29].

### 2.5. Description of the Procedure

Phase (1): Translation and Localization

In the translation and localization process of AtAS, initial approval was obtained from the original designer of the questionnaire, Sofia von Humboldt, by sending an email explaining the purpose of the psychometric evaluation and the preparation of the Persian version of the instrument. The translation process followed the guidelines proposed by Wild et al. (2005) [31] for cross-cultural adaptation of self-report measures. Initially, the original (English) version of the AtAS was independently translated into Persian by the researcher and two Persian speakers, who are proficient in the terminology and translation of gerontology texts. Subsequently, the three translated versions were compared, and the items were reconciled in terms of meaning and concept, resulting in a single preliminary Persian version. To ensure the complete alignment of the Persian translation with the original text and the fluency of the sentences, the preliminary Persian version of the questionnaire was back-translated into English by another translator who was fluent in English and had not previously seen the original questionnaire. The back-translated version was then compared with the original English version, and necessary revisions were made under the supervision of translators and experts (including four PhD-level gerontology specialists). Overall, all items were considered culturally appropriate and understandable, with only minor adjustments made to items 11 and 15 to enhance clarity. Finally, the final Persian version of the AtAS was obtained. (Appendix A)

Phase (2): Psychometric Properties

After translating and preparing the Persian version of The Adjustment to Aging (AtAS), the psychometric properties of the questionnaire, including face and content validity, reliability (internal consistency and stability), construct validity (confirmatory factor analysis), and criterion validity, were evaluated and examined.

### 2.6. Data Analysis

In this study, descriptive statistics, including frequency distribution (percentage), mean, and standard deviation, were used to describe the demographic characteristics of the study sample. For inferential data analysis, considering the normal distribution of data based on skewness and kurtosis tests (in the range of −2 to +2), independent t-tests (to compare the means of two groups), one-way analysis of variance (ANOVA) (to examine differences between means in multiple groups), and Pearson correlation coefficient (to examine the relationship between variables) were used. Data analysis was performed using SPSS (version 22) and AMOS (version 24) software, and the statistical significance level was set at *p* < 0.05 for all tests.

## 3. Results

### 3.1. Data Management and Floor/Ceiling Effects

As part of the data management process, two cases with extreme scores (both scoring the minimum value of 22) were excluded from the analysis, resulting in a final sample size of 328 participants. After their removal, no floor or ceiling effects were observed, as none of the participants reached the minimum (22) or maximum (154) possible scores on the Adjustment to Aging Scale.

### 3.2. Descriptive Results

A total of 328 older adults participated in this study. The mean age of the participants was 69.42 years (SD = 6.8), with an age range of 60 to 90 years. Just over half of the sample were female (56.1%), and the majority were married (69.5%). Regarding education, 67% of participants had a high school diploma or lower level of education.

In terms of employment status, 47.9% were retired, while only 12 individuals were currently employed. Among those employed, 32.7% indicated that financial necessity was the main reason for continuing to work. Concerning economic conditions, more than half of the participants (57.9%) described their financial status as average, while only 1.5% reported it as very good.

With respect to living arrangements, 36% of the participants lived with their spouse and unmarried children. Most participants (77.7%) were residing in their own privately owned homes. Only 22% of the older adults rated their overall health status as excellent. Additionally, 46.6% were covered by both basic and supplementary health insurance plans (Table 1).

The mean (SD) overall Adjustment to Aging scale score was 108.27 (±15.77). The lowest mean score was for the social support (SS) subscale, and the highest mean score was for the Zest and Spirituality (ZS) subscale (Table 2).

The mean scores of adjustment to aging across different demographic variables are presented in Table 3. Adjustment to aging showed a small but significant inverse correlation with age and was significantly higher among men. Higher educational level, more favorable economic status, coverage by both basic and supplementary insurance (compared with basic insurance alone), and better self-rated health were all associated with higher adjustment scores (*p* < 0.05). Differences were also observed across residential areas, marital status, and living arrangements, while no significant associations emerged for home ownership or reason for employment. Detailed statistical results and post hoc comparisons are presented in Table 3 (see Supplementary Materials for full details).

### 3.3. Face Validity

To assess face validity, feedback was obtained from four older adults who met the study’s inclusion criteria as well as from fourteen experts in the fields of gerontology, social work, psychology, and nursing. Participants were asked to evaluate the clarity, comprehensibility, and grammatical appropriateness of the items in the instrument. Based on their feedback, minor modifications were made in consultation with the research team. Specifically, items 11 and 15 were slightly revised to enhance clarity. Overall, all items were reported as understandable and appropriate by both the older adults and the experts, indicating that the instrument demonstrated satisfactory face validity.

### 3.4. Content Validity

The content validity of the questionnaire was evaluating fourteen experts in the fields of gerontology, social work, psychology, and nursing. Following the Waltz and Bausell method [32], the Content Validity Index (CVI) for each item was calculated. The average CVI across all items was 0.94, indicating that the Adjustment to Aging Scale has acceptable content validity.

### 3.5. Construct Validity (Confirmatory Factor Analysis)

**Figure 1:**
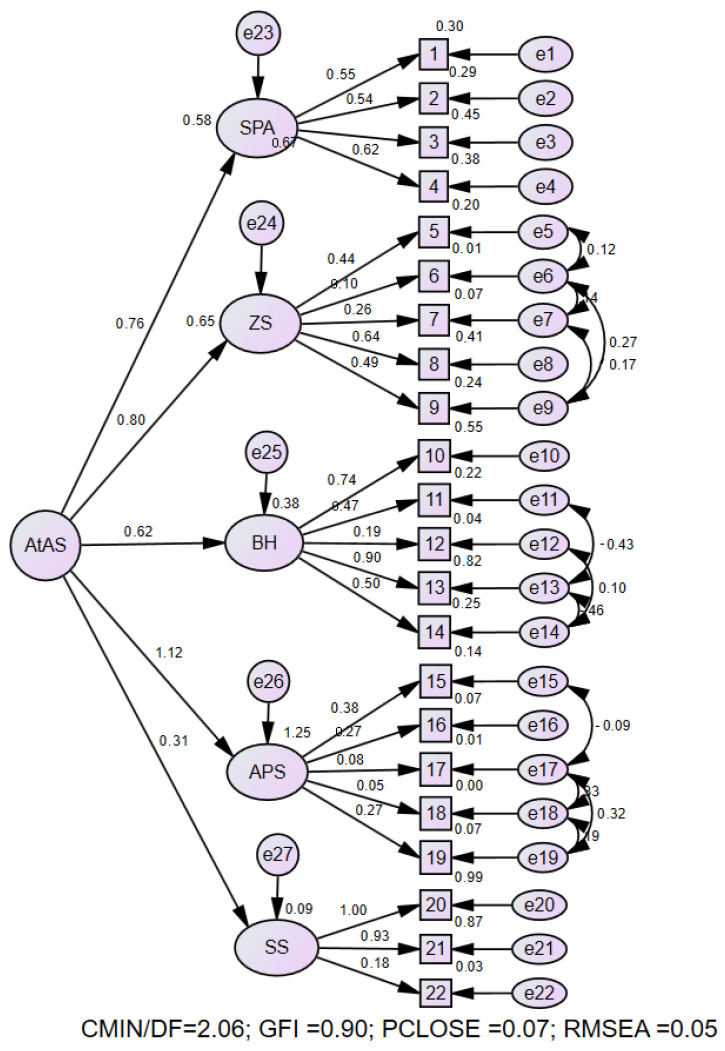
The final measurement model of the research and its parameters using standardized coefficients. SPA: Sense of Purpose and Ambition, ZS: Zest and Spirituality, BH: Body and Health, APS: Aging in Place and Stability, SS: Social Support.

The construct validity of the questionnaire was assessed using a confirmatory factor analysis (CFA). In the initial model, some of the goodness-of-fit indices suggested an inadequate model fit (Table 4). Based on the modification indices, error covariances were added between items belonging to the same latent construct, which is both theoretically and statistically justified. Following these modifications, the model was re-run, and the final model had an acceptable fit. the goodness-of-fit index of the chi-squared per the number of degrees of freedom (χ2/df) was 2.06, the Goodness-of-Fit Index (GFI) was 0.90, the Comparative Fit Index (CFI) was 0.92, the Tucker–Lewis Index (TLI) was 0.91, the Standardized Root Mean Square Residual (SRMR) was 0.003, the Root Mean Square Error of Approximation (RMSEA) was 0.05, and the *p*-value for close fit (PClOSE) was 0.07, indicating an acceptable model fit. The final model is presented in the diagram (Figure 1).

#### 3.5.1. Convergent Validity and Composite Reliability

Convergent validity was assessed using Average Variance Extracted (AVE), and composite reliability (CR) was calculated to evaluate internal consistency. The results indicated acceptable to strong reliability across all five subscales (CR = 0.47 to 0.79), with three dimensions exceeding the standard threshold of 0.70 (Table 5). While most subscales demonstrated acceptable levels of convergent validity, one subscale showed a lower AVE value (0.06), which may be due to cultural differences in how items within that dimension are interpreted. Overall, the scale exhibited sound reliability and partially acceptable convergent validity, suggesting that it is a psychometrically promising instrument. With cultural adaptations and refinement of specific items, the validity of the scale could be further enhanced. Nonetheless, the overall results support the construct validity of the instrument within the target population.

#### 3.5.2. Discriminant Validity

Discriminant validity of the aging adjustment scale was examined using the Fornell–Larcker criterion, which posits that the square root of the Average Variance Extracted (√AVE) for each construct should exceed its correlations with other constructs. The results indicated that most dimensions met this requirement, supporting adequate discriminant validity (Table 6). However, Dimension 4 (Aging in Place and Stability) showed a √AVE of 0.24, which was lower than its correlations with some other dimensions (e.g., 0.29 and 0.38), indicating insufficient discriminant validity for this subscale. This may be attributed to conceptual overlap or cultural factors influencing how stability and place attachment in aging are perceived. Overall, the scale demonstrates satisfactory discriminant validity, though the fourth dimension may benefit from further refinement to improve its construct distinctiveness. Given its lower discriminant validity, the APS (Aging in Place and Stability) subscale may require further conceptual refinement or item re-specification in future studies.

### 3.6. Criterion Validity

To assess criterion validity, the concurrent validity method was used, whereby the correlation between the Adjustment to Aging Scale and WHO 5 well-being index was evaluated. Pearson’s correlation analysis results showed that there is a positive and significant correlation between AtAS and WHO 5 well-being index scores (r = 0.56, *p* < 0.001), indicating that higher levels of adjustment to aging were associated with greater psychological well-being. These findings provide evidence for the criterion validity of the AtAS, indicating that the scale appropriately captures an aspect of aging adjustment that is meaningfully associated with well-being in older adults.

### 3.7. Reliability

The reliability of the instrument was assessed using two methods: internal consistency and test–retest reliability. Internal consistency was evaluated in the full sample of 328 older adults by calculating Cronbach’s alpha coefficient, which was found to be 0.80 for the entire scale, indicating good internal consistency. Test–retest reliability was assessed in a subsample of 30 participants using the intraclass correlation coefficient (ICC). The ICC was calculated based on responses obtained from a two-week interval between the two administrations of the scale. The result showed an ICC of 0.98 with a 95% confidence interval, suggesting excellent temporal stability of the Persian version of the Aging Adjustment Scale (ATAS). Furthermore, the standard error of measurement (SEM) for the total scale was calculated to be 0.12, and the relative measurement error (SEM%) was 14%. These values indicate that the instrument has a low level of measurement error, further supporting the precision and reliability of the scale.

## 4. Discussion

The present study aimed to examine the psychometric properties of the Adjustment to Aging Scale (AtAS) in an Iranian older adult population and to provide a valid Persian-language instrument. Various psychometric aspects of the questionnaire, including face validity, content validity, criterion validity, construct validity, and reliability, were assessed among older adults in Iran in 2024. A total of 328 participants aged 60 years and above took part in the study. The findings indicated that the Persian version of the AtAS demonstrated satisfactory translation quality and acceptable psychometric properties in Iranian older adults. The number of items in the questionnaire is appropriate for older adults, making it easy to administer and quick to complete.

Overall, the descriptive findings of this study indicated that the demographic composition of the participating older adults—regarding age, gender, marital status, socioeconomic status, and common health conditions—was similar to patterns reported in other aging studies in Iran and worldwide [33,34,35,36,37]. However, there were notable differences in education levels across areas, which explain why approximately two-thirds of participants had post-secondary education. This is because the areas with the highest numbers of older adults (Districts 2 and 5) also exhibit higher education rates. In particular, the highest rates of post-secondary education were observed in Districts 2, 4, and 5, with literacy rates of 88.7%, 68.2%, and 78.2%, respectively [28].

In the present study, the mean score of adjustment to aging was 108.27 (SD = 15.77). Analysis of the results revealed that demographic variables such as age, gender, marital status, place of residence, education, economic status, type of insurance, and health status were significantly associated with the level of adjustment among older adults. In this study, increasing age was associated with lower adjustment to aging, which may be due to a decline in resources required for successful adaptation as age advances [38]. One of these resources is both objective and subjective health status, which tends to decrease in older adults with increasing age [39]. Among the most important factors related to mental health are economic status and educational attainment [40]. Consistent with our findings, higher education and better economic status were associated with greater adjustment to aging; previous studies have shown that better economic status and higher educational levels may enhance social participation and influence lifestyle, while also improving access to health resources and services, thereby promoting both physical and mental health [41,42].

Furthermore, our study demonstrated that older adults with better self-reported health reported higher adjustment scores. These findings suggest that cultural capital and financial resources can serve as effective tools for enhancing adjustment to aging. However, a cross-sectional study in Sweden found no significant relationship between economic status and education level with self-rated health [43]. Similarly, Montross et al. reported that most community-dwelling older adults, even those with chronic physical illnesses and certain disabilities, perceived themselves as aging successfully and being well-adjusted [44]. Nevertheless, other studies have emphasized that both physical and mental health are prerequisites for optimal adjustment to aging [45].

Gender also influences adjustment to aging. According to our findings, men scored higher on adjustment to aging compared to women. Similarly, Shi et al. reported that older men experience greater psychological well-being than older women [46]. This difference may stem from variations in social roles, the level of family support, or cultural attitudes toward aging. From the perspective of gender theory, biological differences between men and women, when shaped by cultural, social, and familial contexts, lead to distinct social roles and behavioral patterns. These differences may influence the distribution of responsibilities, access to resources, and adjustment to aging [47]. Furthermore, Social role theory highlights that culturally defined expectations, such as caregiving responsibilities often falling on women, may increase stress and reduce adaptive capacity, whereas men may experience greater autonomy and social recognition, supporting higher adjustment scores [48].

Marital status also showed a significant effect on adjustment. Older adults who were married demonstrated higher adjustment scores than their counterparts. Likewise, Gutierrez-Vega et al. found that marital status is associated with better physical, psychological, and social quality of life and that being married serves as a protective factor against depressive symptoms and mental disorders by providing positive social support [49]. From the perspective of socioemotional selectivity theory, older adults prioritize emotionally meaningful relationships as they age, and being married provides continuous access to close, supportive bonds that can buffer stress and enhance adaptive capacity during aging [50]. In line with social support theory, married individuals also benefit from practical, emotional, and cognitive resources provided by their spouse, further strengthening their ability to cope with age-related challenges [51]. Additionally, life-course theory suggests that marriage contributes to continuity in social roles and the maintenance of stable support networks throughout life, facilitating better adjustment to the transitions and changes associated with later life [52].

The psychometric findings of the present study indicate that the Persian version of the Adjustment to Aging Scale (AtAS) demonstrates satisfactory validity and reliability, with most results aligning closely with the original version conducted by von Humboldt et al. [23]. Face validity assessment, performed by both experts and target respondents, is a well-established approach to ensure item clarity, comprehensibility, and linguistic appropriateness for the intended population [53]. In this study, all items were confirmed in terms of clarity and relevance, with only minor modifications applied to a few items, reflecting adequate face validity of the instrument. Similarly, von Humboldt et al. [23] utilized feedback from groups of older adults and experts to evaluate face validity, reporting the tool as linguistically suitable and comprehensible.

The mean content validity index (CVI) was 0.94, indicating strong content approval of the items from the perspective of experts [54]. The original version [23] also demonstrated content validity across all 22 items, highlighting concordance between the two studies. The high CVI values in both studies affirm the validity of the items in measuring various dimensions of adjustment to aging [32]. These findings are consistent with previous research reporting high content validity for psychometric instruments related to aging [55,56].

In the confirmatory factor analysis (CFA), the five-factor model of the instrument, after modification based on adjustment indices, demonstrated acceptable fit indices including χ^2^/df, GFI, CFI, TLI, SRMR, and RMSEA, indicating a good fit between the model and the data. These results conform to international standards for CFA [57] and confirm the construct validity of the scale. The findings are consistent with the factor structure reported in the original instrument validation by von Humboldt et al. [23], reflecting the stability of the factor structure across different cultures and languages.

Convergent validity was evaluated using the average variance extracted (AVE) and composite reliability (CR). Three dimensions of the scale showed CR values above 0.70, indicating strong internal consistency, and most dimensions exhibited acceptable AVE values. However, one dimension had an AVE below 0.5, which may reflect cultural differences in the perception of its items. Previous studies suggest that while the overall factor structure remains stable, certain dimensions require careful attention in cross-cultural translation and emphasize the necessity of cultural adaptation for psychometric tools [58,59]

Regarding discriminant validity, results showed that for most dimensions, the square root of the AVE exceeded the correlations with other constructs, indicating adequate construct distinctiveness. Nevertheless, the fourth dimension (Stability and Staying in the Living Environment) exhibited a lower square root of AVE compared to some correlations, suggesting relative weakness in construct discrimination and potential need for conceptual revision. This finding aligns with the psychometric results from the original study [23], where the “Place Stability” dimension showed the least discriminant validity. Such issues are common in translated and culturally adapted instruments [60] and may stem from cultural differences or varied interpretations among older adults regarding the concept of place stability.

Criterion validity assessment revealed a significant positive correlation between the Adjustment to Aging Scale (AtAS) and the WHO-5 Well-Being Index (r = 0.56), indicating that adjustment to aging is associated with psychological well-being among older adults. In the original version [23], the AtAS demonstrated significant correlations with the Satisfaction with Life Scale (SwLS) and the Positive and Negative Affect Schedule (PANAS). These findings align with previous research reporting relationships between psychological adjustment to aging and subjective well-being [61], supporting the cross-cultural validity of the instrument in predicting mental health-related outcomes.

Finally, reliability analysis showed that the overall Cronbach’s alpha coefficient was 0.80, and the Intraclass Correlation Coefficient (ICC) for test–retest reliability was 0.98, reflecting satisfactory internal consistency and excellent temporal stability. Additionally, the low standard error of measurement (SEM = 0.12) indicates high precision of the instrument over time. von Humboldt’s study [23] similarly reported a Cronbach’s alpha of 0.89 and an ICC of 0.98. Therefore, it can be concluded that the Persian version of the scale demonstrates reliability comparable to the original version, with results consistent with similar psychometric studies in older adult populations [62,63].

### Strengths and Limitations

One of the key strengths of this study is that it was conducted in Tehran, the capital of Iran, which is characterized by substantial cultural diversity, and included a relatively large sample size (n = 328). These factors collectively enhance the generalizability of the findings. However, although the Persian version of the AtAS demonstrated strong psychometric properties, the Aging in Place and Stability (APS) dimension showed weaker convergent and discriminant validity, indicating that further refinement may be necessary for optimal cultural adaptation. Another limitation is that the Persian AtAS was not formally culturally adapted; however, the original scale was designed for cross-cultural use, and its translation and psychometric validation were successfully conducted. A further limitation is that the proportion of older adults attending each health center is unknown. Moreover, the study sample included a relatively high proportion of literate participants, which should be considered when generalizing the findings to populations with lower literacy levels.

## 5. Conclusions

This study confirms that the Persian version of the Adjustment to Aging Scale (AtAS) is a valid and reliable tool for assessing aging adjustment in Iranian older adults. The scale demonstrated strong validity across multiple domains and excellent reliability, consistent with the original version. Its stable five-factor structure and meaningful correlations with demographic and well-being indicators support its cultural relevance and practical utility. Despite minor limitations in one dimension, the Persian AtAS offers a valuable instrument for psychological research and clinical assessment in Iran, enabling better understanding and support of successful aging. Future studies should consider longitudinal evaluation and, more specifically, conduct comprehensive cultural refinement. This may include engaging expert panels of cultural insiders to review item content, testing measurement invariance across diverse subgroups, and iteratively revising or adapting items to address identified discrepancies. Future research is also recommended to examine the Persian AtAS in various Iranian settings beyond Tehran and to follow participants over time to capture changes in adjustment. Such efforts would further enhance the applicability and validity of the Persian AtAS across different cultural and socio-economic contexts.

## Figures and Tables

**Table 1:** Demographic characteristics of the participants (N = 328).

Variables		Frequency (%)
**Age**	Mean (SD)	69.42 (±6.8)
**Gender**	Male Female	144 (43.9%) 184 (56.1%)
**Marital Status**	Never Married Married Widowed divorced	11 (3.4%) 288 (69.5%) 69 (21%) 20 (6.1%)
**Education Status**	Illiterate Literate (no formal education) Primary Lower Secondary Upper Secondary Diploma University Education	24 (7.3%) 7 (2.1%) 36 (11%) 26 (7.9%) 17 (5.2%) 110 (33.5%) 108 (33%)
**Employment Status**	Employment Full-Time Employment Part-Time Homemaker Retired Disable/Unable to Work	18 (5.5) 21 (6.4) 127 (38.7) 157 (47.9) 5 (1.5)
**Reason for Employment**	Financial necessity Habit and leisure Both reasons	17 (32.7) 7 (13.5) 28 (53.8)
**Economic Status**	Very good Good Average Poor Very poor	5 (1.5) 70 (21.3) 190 (57.9) 46 (14) 17 (5.2)
**Home Ownership**	Owned Rented/Mortgaged Child’s Home Relative’s or friend’s Home	255 (77.7) 66 (20.1) 5 (1.5) 2 (0.6)
**Living Arrangements**	Living alone With spouse only With spouse and unmarried children With spouse and married children Without spouse, with unmarried children Without spouse, with married children With relatives other	49 (14.9) 102 (31.1) 118 (36) 6 (1.8) 35 (10.7) 7 (2.1) 8 (2.4) 3 (0.9)
**general Health Status**	Excellent Very good Good Fair poor	22 (6.7) 14 (4.3) 126 (38.4) 139 (42.4) 27 (8.2)
**Type of Insurance Coverage**	Basic insurance Supplementary insurance Both none	80 (24.4) 60 (18.3) 153 (46.6) 35 (10.7)

**Table 2:** Descriptive statistics of Adjustment to Aging across subscales.

Dimension	Score Range	Mean	SD
**SPA**	4–28	18.70	5.21
**ZS**	5–35	26.32	4.50
**BH**	5–35	24.42	5.71
**APS**	5–37	22.90	5.05
**SS**	3–21	15.84	4.11
**Total**	22–154	108.27	15.77

**Table 3:** Adjustment to Aging Scores across sociodemographic subgroups.

Variables		Mean (SD)	T/F Statistic	*p*-Value	Post hoc Tukey Tests
**Age**			R = −0.16	0.002 *	-
**Gender**	1. Male 2. Female	110.29 (15.54) 106.57 (15.73)	t (326) = 2.13	0.03 *	-
**Marital Status**	1. Never Married 2. Married 3. Widowed 4. divorced	102.09 (14.69) 110.74 (15.14) 100.72 (14.93) 108.45 (17.59)	F (3, 324) = 8.24	*p* < 0.001 *	2 > 3, *p* < 0.05
**Education Status**	1. Illiterate 2. Literate (no formal education) 3. Primary 4. Lower Secondary 5. Upper Secondary 6. Diploma 7. University Education	92.04 (15.75) 109.28 (17.31) 110.97 (17.28) 110.38 (13.84) 106.05 (14.13) 106.47 (15.21) 112.38 (13.95)	F(6, 321) = 6.66	*p* < 0.001 *	3 > 1, *p* < 0.05 4 > 1, *p* < 0.05 6 > 1, *p* < 0.05 7 > 1, *p* < 0.05
**Employment Status**	1. Employment Full-Time 2. Employment Part-Time 3. Homemaker 4. Retired 5. Disable/Unable to Work	114.16 (14.13) 105.47 (13.07) 106.18 (15.97) 110.01 (15.20) 92.80 (26.95)	F(4, 323) = 3.12	0.01 *	-
**Reason for Employment**	1. Financial necessity 2. Habit and leisure 3. Both reasons	107.41 (14.49) 111.57 (17.36) 109.82 (13.61)	F(2, 49) = 0.25	0.77	-
**Economic Status**	1. Very good 2. Good 3. Average 4. Poor 5. Very poor	124.20 (5.89) 113.74 (14.79) 108.39 (15.36) 99.60 (13.66) 101.88 (18.05)	F(4, 323) = 8.25	*p* < 0.001 *	1 > 4, *p* < 0.05 1 > 5, *p* < 0.05 2 > 4, *p* < 0.05 2 > 5, *p* < 0.05 3 > 4, *p* < 0.05
**Home Ownership**	1. Owned 2. Rented/Mortgaged 3. Child’s Home 4. Relative’s or friend’s Home	108.82 (15.42) 105.78 (16.53) 116 (12.56) 90 (26.87)	F(3, 324) = 1.96	0.11	-
**Living Arrangements**	1. Living alone 2. With spouse only 3. With spouse and unmarried children 4. With spouse and married children 5. Without spouse, with unmarried children 6. Without spouse, with married children 7. With relatives 8. other	104.22 (16.60) 110.70 (15.40) 110.23 (15.21) 100.16 (10.26) 101.62 (14.41) 92.57 (13.62) 114.25 (13.18) 121.66 (14.97)	F(7, 320) = 3.88	*p* < 0.001 *	-
**Area of residence**	1. Region 2 2. Region 4 3. Region 5 4. Region 14 5. Region 15	111.65 (14.49) 107.28 (17.19) 104.52 (15.95) 107.88 (13.57) 111.10 (15.13)	F(4, 323) = 2.67	0.03 *	1 > 3, *p* < 0.05
**general Health Status**	1. Excellent 2. Very good 3. Good 4. Fair 5. poor	121.18 (8.48) 121 (8.73) 113.69 (12.79) 103.35 (14.96) 90.33 (14.46)	F(4, 323) = 29.52	*p* < 0.001 *	1 > 4, *p* < 0.05 1 > 5, *p* < 0.05 2 > 4, *p* < 0.05 2 > 5, *p* < 0.05 3 > 4, *p* < 0.05 3 > 5, *p* < 0.05 4 > 5, *p* < 0.05
**Type of Insurance Coverage**	1. Basic insurance 2. Supplementary insurance 3. Both 4. none	104 (15.29) 108.90 (16.32) 110.45 (15.05) 106.80 (17.20)	F(3, 324) = 3.14	0.02 *	3 > 1, *p* < 0.05

Items marked with "*" were statistically significant.

**Table 4:** Goodness-of-fit indices for the measured model of the Adjustment to Aging Scale.

Model	**X^2^/DF**	**GFI**	RMSEA	PCLOSE	CFI	TLI	SRMR
**Recommended value**	<3	≥0.9	<0.1	>0.05	≥0.9	≥0.9	<0.08
**AtAS**	2.499	0.86	0.06	0.000	0.88	0.86	0.06
**AtAS** **(corrected)**	2.06	0.90	0.05	0.07	0.92	0.91	0.003

X^2^/DF: Chi-square test, GFI: goodness-of-fit index, RMSEA: root mean square error of approximation, PCLOSE: *p*-value for close fit.

**Table 5:** Psychometric Properties: Convergent Validity (AVE) and Reliability (CR) of the Scale Dimensions.

Dimensions of AtAS	**SPA**	**ZS**	**BH**	**APS**	**SS**
**AVE**	0.36	0.18	0.37	0.06	0.62
**CR**	0.70	0.47	0.71	0.19	0.79

AVE: Average Variance Extracted, CR: Composite Reliability.

**Table 6:** Discriminant Validity Assessment (Fornell- Larcker Criterion).

	√AVE	SPA	ZS	BH	APS	SS
**SPA**	0.60	-	0.36	0.38	0.17	0.21
**ZS**	0.42	0.36	-	0.19	0.32	0.12
**BH**	0.61	0.38	0.19	-	0.29	0.29
**APS**	0.24	0.17	0.32	0.29	-	0.19
**SS**	0.79	0.21	0.12	0.29	0.19	-

## Data Availability

Data supporting the findings of this study are available upon reasonable request to the corresponding author.

## Supplementary Materials

The following supporting information can be downloaded at: https://www.mdpi.com/article/10.3390/geriatrics10060149/s1, detailed demographic data and statistical results corre-sponding to Table 3 have been included in the supplementary materials.

## Abbreviations

The following abbreviations are used in this manuscript:

**AtAS**	**Adjustment to Aging Scale**
CVI	Content Validity Index
CFA	Confirmatory Factor Analysis
GFI	Goodness-of-Fit Index
RMSEA	Root Mean Square Error of Approximation
PCLOSE	P-value for Close fit

## Appendix A

### Persian Version of the Adjustment to Aging Scale (AtAS)

**خیلی موافقم**	**موافقم**	**تاحدودی موافقم**	**نظری ندارم**	**تاحدودی مخالفم**	**مخالفم**	**خیلی مخالفم**	**بر اساس موقعیت خود در یک سال گذشته پاسخ دهید**	**ردیف**
							فعال هستم و در حوزه مورد علاقه ام فعالیت میکنم	1
							کنجکاو هستم و به یادگیری علاقه دارم	2
							خلاق هستم و چیزهای جدیدی درست میکنم	3
							اثر گذار هستم و برای آینده تلاش میکنم	4
							خنده رو، شوخ طبع و اهل تفریح هستم	5
							به دین و معنویت اعتقاد دارم و آدم معنوی هستم	6
							تغییرات زندگی را میپذیرم	7
							از عمر خود بهترین استفاده را میکنم	8
							نسبت به آینده احساس آرامش دارم	9
							سالم هستم و درد یا بیماری ندارم	10
							بیرون از منزل ورزش میکنم و فعالیت بدنی دارم (پیاده روی و ...)	11
							با اصول خودم زندگی میکنم و مستقل هستم	12
							به دارو یا درمان خاصی وابستگی ندارم	13
							از بدن و ظاهر خود راضی هستم	14
							بیرون از خانه تحرک و فعالیت دارم (خرید و....)	15
							همسایه های حامی دارم	16
							آب و هوای محل زندگی ام خوب و سالم است	17
							بیرون از منزل احساس امنیت میکنم	18
							ثبات و آسایش اقتصادی دارم	19
							با همسرم (شریک زندگی ام) صمیمی هستم	20
							همسر (همراه) خوبی دارم	21
							برای خانواده ام عزیز هستم	22
