# Supplementary material for: Psychometric Properties of the Adjustment to Aging Scale (Atas) in Iranian Older Adults
Source: Geriatrics (Basel). 2025 Nov 10;10(6):149. doi: 10.3390/geriatrics10060149 (PMC12641847; doi:10.3390/geriatrics10060149)
Supplement: Supplementary file 1 [file geriatrics-10-00149-s001.zip › geriatrics-3847819-supplementary.pdf]

Supplementary to Table 3. Detailed demographic characteristics of study participants and post hoc comparisons for adjustment to aging

Results from Pearson correlation analysis showed a significant inverse correlation between age and adjustment to aging ($r = -0.16$, $p = 0.002$). An independent t-test indicated a significant difference in adjustment scores between men and women, with men reporting significantly higher adjustment than women ($t(362) = 2.13$, $p = 0.03$).

One-way ANOVA revealed significant associations between adjustment to aging and several demographic variables. Participants' area of residence was significantly related to adjustment ($F(4, 323) = 2.67$, $p < 0.05$); post hoc Tukey tests indicated that older adults living in District 2 had significantly higher adjustment scores than those in District 5 ($p < 0.05$). Marital status was also significantly associated with adjustment ($F(3, 324) = 8.24$, $p < 0.05$), and married individuals showed significantly higher adjustment compared to those who were divorced ($p < 0.05$). Educational level showed a significant relationship with adjustment ($F(6, 321) = 6.66$, $p < 0.05$). Tukey post hoc analysis revealed that individuals with elementary, secondary, high school, or university education had significantly higher adjustment scores than those who were illiterate ($p < 0.05$). Employment status was also significant ($p < 0.05$), although pairwise comparisons did not reach significance ($p = 0.053$), indicating a potential trend for higher adjustment among full-time employed older adults compared to those who were disabled.

Living arrangements were significantly related to adjustment ($p < 0.05$); although post hoc results did not show statistically significant differences ($p = 0.052$), a trend was observed in which older adults living with their married children without a spouse had lower adjustment compared to those living only with their spouse. Economic status showed a significant association with adjustment ($F(4, 323) = 8.25$, $p < 0.05$). Older adults who reported their financial situation as "very good" or "good" had significantly higher adjustment than those with "poor" or "very poor" status ($p < 0.05$). Even those with an "average" financial status showed significantly higher adjustment than those with "poor" economic status ($p < 0.05$).

Insurance coverage was also significantly related to adjustment ($F(3, 324) = 3.14$, $p < 0.05$), with those covered by both basic and supplementary insurance reporting higher adjustment than those with only basic insurance ($p < 0.05$). No significant differences were found for reason of employment or home ownership ($p > 0.05$). However, self-rated health status was significantly associated with adjustment. Individuals who rated their health as excellent, very good, or good reported significantly higher adjustment than others ($p <$

0.05), and those who rated their health as “somewhat good” also had significantly higher adjustment than those who rated their health as poor ($p < 0.05$).
